# Comparing a pre-defined versus deep learning approach for extracting brain atrophy patterns to predict cognitive decline due to Alzheimer’s disease in patients with mild cognitive symptoms

**DOI:** 10.1186/s13195-024-01428-5

**Published:** 2024-03-19

**Authors:** Ida Arvidsson, Olof Strandberg, Sebastian Palmqvist, Erik Stomrud, Nicholas Cullen, Shorena Janelidze, Pontus Tideman, Anders Heyden, Karl Åström, Oskar Hansson, Niklas Mattsson-Carlgren

**Affiliations:** 1 Centre for Mathematical Sciences, Lund University, Lund, Sweden; 2 Clinical Memory Research Unit, Department of Clinical Sciences, Malmö, Lund University, Lund, Sweden; 3 Memory Clinic, Skåne University Hospital, Malmö, Sweden; 4 Wallenberg Center for Molecular Medicine, Lund University, Lund, Sweden; 5 Department of Neurology, Skåne University Hospital, Lund, Sweden

**Keywords:** Alzheimer’s disease, Cognitive decline, Deep learning

## Abstract

**Background:**

Predicting future Alzheimer’s disease (AD)-related cognitive decline among individuals with subjective cognitive decline (SCD) or mild cognitive impairment (MCI) is an important task for healthcare. Structural brain imaging as measured by magnetic resonance imaging (MRI) could potentially contribute when making such predictions. It is unclear if the predictive performance of MRI can be improved using entire brain images in deep learning (DL) models compared to using pre-defined brain regions.

**Methods:**

A cohort of 332 individuals with SCD/MCI were included from the Swedish BioFINDER-1 study. The goal was to predict longitudinal SCD/MCI-to-AD dementia progression and change in Mini-Mental State Examination (MMSE) over four years. Four models were evaluated using different predictors: (1) clinical data only, including demographics, cognitive tests and *APOE* ε4 status, (2) clinical data plus hippocampal volume, (3) clinical data plus all regional MRI gray matter volumes (*N* = 68) extracted using FreeSurfer software, (4) a DL model trained using multi-task learning with MRI images, Jacobian determinant images and baseline cognition as input. A double cross-validation scheme, with five test folds and for each of those ten validation folds, was used. External evaluation was performed on part of the ADNI dataset, including 108 patients. Mann-Whitney U-test was used to determine statistically significant differences in performance, with *p*-values less than 0.05 considered significant.

**Results:**

In the BioFINDER cohort, 109 patients (33%) progressed to AD dementia. The performance of the clinical data model for prediction of progression to AD dementia was area under the curve (AUC) = 0.85 and four-year cognitive decline was R^2^ = 0.14. The performance was improved for both outcomes when adding hippocampal volume (AUC = 0.86, R^2^ = 0.16). Adding FreeSurfer brain regions improved prediction of four-year cognitive decline but not progression to AD (AUC = 0.83, R^2^ = 0.17), while the DL model worsened the performance for both outcomes (AUC = 0.84, R^2^ = 0.08). A sensitivity analysis showed that the Jacobian determinant image was more informative than the MRI image, but that performance was maximized when both were included. In the external evaluation cohort from ADNI, 23 patients (21%) progressed to AD dementia. The results for predicted progression to AD dementia were similar to the results for the BioFINDER test data, while the performance for the cognitive decline was deteriorated.

**Conclusions:**

The DL model did not significantly improve the prediction of clinical disease progression in AD, compared to regression models with a single pre-defined brain region.

**Supplementary Information:**

The online version contains supplementary material available at 10.1186/s13195-024-01428-5.

## Introduction

Alzheimer’s disease (AD) affects millions of individuals worldwide. AD dementia is characterized by a prolonged prodromal phase in which amyloid pathology accumulates in the brain before cognitive decline starts [1]. The subsequent onset of tau pathology and atrophy tracks more closely with occurrence of symptoms in the early phase of the disease in which individuals experience only subjective cognitive decline (SCD), or cognitive decline that qualify for mild cognitive impairment (MCI) [1, 2]. However, despite this understanding of the pathophysiological cascade of AD, it is not straight forward which individuals, who are present in a clinical setting with SCD/MCI, will progress to AD dementia (early AD) versus those that remain with stable SCD/MCI or develop other dementias (non-AD) [3–5]. It is also often unclear at which rate individuals with SCD/MCI will continue to decline cognitively, particularly due to varying biological resiliency to AD pathology at the individual level [6]. With the recent breakthroughs in disease-modifying treatments (DMT) against AD [7, 8], it may be possible to alter the course of disease in patients with SCD/MCI due to AD. Given the heterogeneity of the SCD/MCI population, it is now urgent to bring forward methods that can guide physicians when making decisions about which patients that are most likely to benefit from receiving DMTs targeting AD pathology.

To improve the prognosis of AD-related cognitive changes, artificial intelligence (AI) could be useful. Features from clinical data and different biomarker modalities can automatically be extracted and combined, to guide in the discrimination between individuals who will remain with SCD/MCI and those who will be diagnosed with AD dementia. One of the most common imaging modalities used for this task is magnetic resonance imaging (MRI). Several previous studies have utilized AI and MRI for this, including for example [9–24]. As can be seen in the review by Grueso et al. [25], the AI methods used vary but often include the support vector machine. However, in more recent studies the usage of deep learning (DL) and convolutional neural networks (CNN) have become frequent thanks to larger datasets and increased computational power. With DL it is possible to extract complex features from a large amount of data, such as a 3D MRI image. A review of publications regarding AD dementia detection using DL is given in [26], showing that MRI is the most widely available and used biomarker for this task, but a variety of DL models are used, including both voxel-based, slice-based, patch-based and region of interest-based. Despite this development in methods, there is only a few studies [14, 22] that objectively compare the performance of AI methods with more intuitive methods, such as logistic or linear regression models with a restricted number of predictors of cognitive decline in the early stages of AD. In SCD/MCI patients, there is also a lack of studies for prediction of longitudinal cognitive decline using commonly used continuous measures of cognition, rather than progression to AD dementia as an outcome.

The main goal of the present study was to perform an unbiased comparison between models utilizing different baseline information for prediction of future cognitive decline in SCD/MCI patients. This is done to understand which variables hold the most information and which method is the most accurate. Two different predictions were done – progression from SCD/MCI to AD dementia within four years (binary outcome) and four-years Mini Mental State Examination (MMSE) slope (continuous outcome). The variables evaluated were demographic information, baseline cognition based on cognitive tests, *APOE* genotype, predefined volumetric variables, and MRI images. The models used were logistic and linear regression, random forest as well as a DL model consisting of a three-dimensional (3D) CNN. Understanding and evaluating the clinical usefulness of these models have rarely been done in an independent and prospective manner. The present study aims to answer whether most of the prognostic information about cognitive decline in an MRI scan is contained in features of the brain (1) which can be obtained through volumetric analysis of pre-specified brain regions, or (2) which can only be obtained through an AI model identifying novel and previously unspecified patterns of brain structure. In this sense, the present study can provide insight as to which level of abstraction prognostic information for AD dementia progression is found.

## Methods

### Cohort description – BioFINDER

Individuals with SCD or MCI were included from the Swedish BioFINDER-1 study (clinical trial no. NCT01208675). The study protocol is described in detail before [27]. Briefly, the consecutively recruited participants in BioFINDER-1 are aged between 60 and 80 years, perform ≥ 24 points on the MMSE, and have been referred to any of the participating memory clinics due to cognitive complaints. A neuropsychological assessment including a comprehensive test battery was used to classify participants as SCD or MCI as previously described [28]. All patients with MCI were classified based on the DSM-5 criteria for MCI [29]. Note however, that for this study the SCD and MCI groups were analyzed together, since the aim of the project was to develop methods that would be useful for longitudinal predictions in an unselected group of patients with cognitive complaints, prior to dementia. Exclusion criteria were cognitive impairment that could be better accounted for by another non-neurodegenerative condition, severe somatic disease, and current alcohol or substance abuse. Only patients with available baseline MRI scans and longitudinal cognitive follow-up of at least four years from baseline were included here. Demographic information (age, sex, education), *APOE* ε4 carriership status (negative/positive) and baseline cognition (MMSE score and Alzheimer’s Disease Assessment Scale [ADAS] delayed word recall) were collected for all individuals.

To guarantee reproducibility and robustness of the models, a double cross-validation scheme was used, such that the data from all individuals could be utilized for both training, validation and test. A five-fold division into development set (80%, used for training and validation) and test set (20%) was used. A stratified random split was used, such that there was no significant difference between development and test sets in the distribution of diagnosis, age, education, sex, or *APOE* status. The stratified random split was produced by randomly splitting the dataset until a division fulfilling the constraints was obtained. For the development sets a 10-fold cross-validation was used, where the folds were drawn such that the same ratio of early AD and non-AD was obtained for each fold, but otherwise randomly. Similarly to the division into test and development, the stratified random split was produced by randomly splitting the dataset until a division fulfilling the constraints was obtained. The double cross-validation scheme was chosen to get robustness and good estimates of the uncertainties of the results. An overview of the demographics for the cohort can be seen in Table 1.

### Cohort description – ADNI

A sub cohort of the Alzheimer’s Disease Neuroimaging Initiative (ADNI) dataset was used for independent evaluation. All participants included had MCI at baseline, at least four-year longitudinal cognitive follow-up from baseline and either MCI or AD dementia after four years. The same variables as included from the BioFINDER cohort where included, including MRI images. An overview of the demographics for the cohort can be seen in Table 1.

**Table 1:** Demographic of patients included in the study

	BioFINDER-1 cohort		ADNI cohort	
	Non-AD	Early AD	Non-AD	Early AD
No. of subjects	332		108	
	223 (67.2%)	109 (32.8%)	85 (78.7%)	23 (21.3%)
MCI	92 (41.2%)	78 (71.6%)	85 (100%)	23 (100%)
Female	106 (47.5%)	51 (46.8%)	27 (31.8%)	10 (43.5%)
Age (years)	70.2 ± 5.5	72.1 ± 4.7	70.9 ± 7.0	74.0 ± 4.9
Education (years)	12.0 ± 3.7	11.8 ± 3.5	16.1 ± 2.6	16.1 ± 2.9
Baseline MMSE score	28.2 ± 1.7	27.1 ± 1.7	28.3 ± 1.5	25.9 ± 2.2
*APOE ε4*	0.4 ± 0.6	1.0 ± 0.7	0.5 ± 0.7	0.9 ± 0.7
ADAS-cog delayed recall (no of errors)	4.2 ± 2.4	6.7 ± 2.1	4.1 ± 2.2	6.6 ± 2.4
Hippocampal volume (mm^3^)	3339 ± 480	2927 ± 415	3592 ± 447	3130 ± 483
Intracranial volume (cm^3^)	1136 ± 153	1114 ± 107	1502 ± 134	1440 ± 155
Aβ-positive	25%	100%	29%	82%

Values are n (%) or mean ± standard deviation. Aβ-status defined by CSF Aβ42/Aβ40 in BioFINDER-1 (available in N=238) and by 18F-florbetapir in ADNI (available in N=85). The BioFINDER cohort was used for both training, validation and testing, in a double cross-validation setting. The ADNI cohort was used for external evaluation

### Study outcomes

There were two outcomes of interest. The primary binary outcome was four-year progression to AD dementia, where a clinical diagnosis of AD dementia during the four years follow-up was considered as a progression. Clinical status of AD dementia was evaluated according to the DSM-5 criteria for major neurocognitive disorders and recorded at each visit by a senior neuropsychologist and experienced memory disorder specialist. Additionally, a diagnosis of AD dementia was only used if the participant had an abnormal cerebrospinal fluid profile consistent with AD pathological change. The diagnostic process is described in detail in [27]. The primary continuous outcome was four-year cognitive decline as measured by the change from baseline in the MMSE score. MMSE is a measure of global cognition, and ranges from 0 to 30 where lower scores indicate worse cognition. Four-year cognitive decline was measured by fitting linear regression models for each individual separately using all available follow-up data within four years from baseline, then extracting the estimated regression slope.

### MRI procedures

T1-weighted MRI was performed on a 3T Skyra MRI scanner (Siemens Healthineers, Erlangen, Germany) producing a high-resolution anatomical MP-RAGE image (TR = 1950 ms, TE = 3.4 ms, 1 mm isotropic voxels, 178 slices). The MRI images were minimally processed using skull stripping, bias correction, and normalization to MNI152 template space [30] using ANTS [31] (normalized using a multi-resolution level [shrink factors = 8, 4, 2, 1, and smoothing sigmas = 3, 2, 1, 0] approach with rigid [mutual information metric 32 bins, regular sampling 25%], affine [mutual information metric 32 bins, regular sampling 25%] and SyN [affine + deformable, cross correlation metric, search radius = 4, full sampling]). Cortical reconstruction and volumetric segmentation were performed with the FreeSurfer image analysis pipeline, as described previously [32]. The Jacobian determinant (JD) images where computed based on the anatomical MRIs non-linear warp to template space and quantify the local deformations, wherein reduced brain matter and atrophy are gauged. Thus, the MRI images are used in native space, as a normalization to MNI space could erase the crucial information, while the JD images are in MNI template space as they map out the expansion/contraction of voxels relative a normal brain and are expected to add some information to the MRI in the form of tissue loss and atrophy relative a normal template brain.

### Sets of predictors

**Fig. 1:**
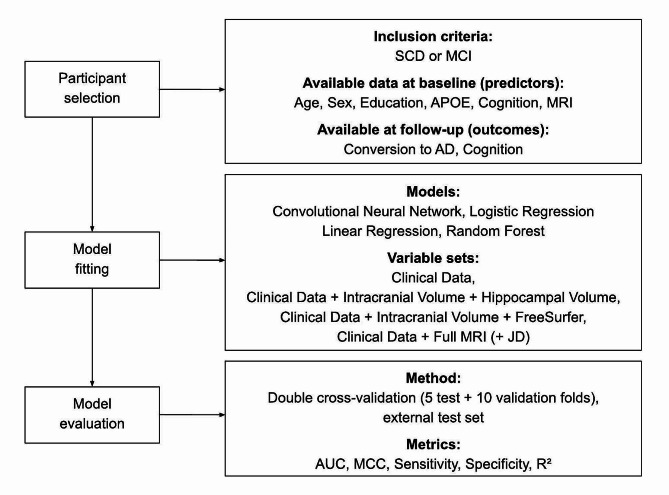
Overview of study. The study consisted of three parts: participant selection, model fitting, and model evaluation

Several *a priori* measurements are related to change in cognition, wherefor we investigated several different types of data and models, see Fig. 1. The first model [“Clinical data model”] utilized readily available demographics information (age, sex, and education), MMSE score, ADAS delayed word recall [33] and *APOE* status. The second model [“Hippocampal volume model”], used hippocampal volume (average of left and right hemisphere) as well as intracranial volume added to the clinical data model. The third model [“FreeSurfer model”], used regional brain volumes from the FreeSurfer pipeline together with intracranial volume added to the clinical data model. The fourth model [“DL model”] used whole brain MRI and JD images along with the clinical data variables in a CNN model. The different models are described in more detail below.

### Basic and Volumetric models

For the clinical data model and the hippocampal volume model we trained logistic regression models for prediction of progression to AD dementia and linear regression models for prediction of longitudinal cognitive decline. For the FreeSurfer models, random forest was used.

For all of the models the features were standardized by removing the mean and scaling to unit variance. The models were optimized using the Scikit-learn library (v. 0.22) in Python (v. 3.5) [34]. For the random forest models, the random forest classifier was used for prediction of progression to AD dementia and the random forest regressor was used for prediction of longitudinal cognitive decline, both with default parameters.

### Deep learning models

CNNs work by learning a successively more complex representation of images across its increasing layers, where the earliest layers closest to the input image are activated by simple shapes such as edges, followed by more complex structures. This method of creating an increasing complex visual representation is similar to how the brain’s visual cortex processes images. We used the CNN architecture suggested by Spasov et al. [14], which is a parameter-efficient network, reducing the risk of overfitting when using small datasets, and has previously been proven successful [13, 14]. We modified the network slightly for our settings, see Supplementary Fig. 1. The model utilizes both the MRI image, the JD image and the clinical data. The main modification done compared to [14] was to train the network for new tasks using multi-task learning [35], which reduces the risk of overfitting the model.

When using multi-task learning the network is trained for several tasks simultaneously, which for example can be beneficial when the dataset used is limited in size and thus the risk of overtraining is high. The multi-task learning was implemented by using three output layers, one for each of the tasks (i) discrimination for four-year progression to AD with sigmoid activation and class weighted categorical cross-entropy loss ${L}_{1}$, (ii) prediction of four-year cognitive decline measured with MMSE slope with linear activation and mean-squared-error loss ${L}_{2}$, and (iii) prediction of hippocampal volume with linear activation and mean-squared-error loss ${L}_{3}$. Thus, each training example was used for all three tasks and the total loss function $L$ was a weighted sum of the three individual ones with weights ${w}_{1},{w}_{2},{w}_{3}$,

1 $$L={w}_{1}{L}_{1}+{w}_{2}{L}_{2}+{w}_{3}{L}_{3} .$$

Depending on which task was the main one, the weighting of the different individual losses was modified. For the model discriminating four-year progression to AD, we used ${w}_{1}=1$ and ${w}_{2}={w}_{3}=0.025$ which was found being a good configuration by testing values in the range 0-0.1, see Supplementary Table 1. Similarly, we used ${w}_{2}=1$ and ${w}_{1}={w}_{3}=0.025$ for the model predicting MMSE slope, see Supplementary Table 2.

The size of our MRI images and JDs differed slightly from the sizes used in the work by Spasov et al. [14]. To be able to use the same network architecture, the MRIs were cropped, and the JDs were padded with zeros. The MRI images were rescaled to have voxel values in range [-1, 1], by dividing by 0.5 times the largest voxel value in the entire MRI set and subtracting 1. No normalization of the JD images was done. The clinical data was all individually rescaled to have values in range [0,1], by removing the minimum value and dividing by the difference between the maximum and minimum value. The normalization techniques were based on the ones used in [14], but slightly modified based on performance on validation data.

Similar to the settings used in [14], the network was trained for 50 epochs using the Adam optimizer with the same learning rate scheduler. The model was implemented based on the code provided by [14] but with the final layers modified to be able to use multi-task learning. The implementation was done in Python (version 3.8) using the Keras library [36] with TensorFlow [37] as backend and trained on a Nvidia Tesla V100 graphics card with 32GB VRAM.

### Statistical analysis

The primary analysis involved the models described above. A sensitivity analysis was also performed looking at the effect of including the MRI image only, the JD image only, or both in the DL model. Due to the training of each DL model taking several days, only one test fold was used in the sensitivity analysis. To improve model interpretability, canonical patterns of brain atrophy for the DL model were identified. Brain atrophy patterns are individualized in nature, so the block occlusion method was followed whereby parts of the image were systematically set to 0 and model performance was evaluated with these images and compared to images without any occlusion. Performing this procedure by systematically blacking out all parts of the image at different trials results in a whole brain atrophy pattern where it is assumed that regions whose blacking out results in a large decrease in model performance must be important to the DL model [38, 39]. This was done for five non-AD and five early AD individuals. The average of the results from the ten folds and ten individuals were used for the final illustrations.

The performance metric of interest for the binary outcome of four-year progression to AD dementia was area under the receiver operating characteristics curve (AUC) and Matthews correlation coefficient (MCC). For the continuous outcome of four-year cognitive decline, the performance metric of interest was R^2^.

The model fitting procedure involved first performing 10-fold cross validation on the development set, where the training set was the part of the data used to determine model parameters and the validation set was the part of the development set which was held out during cross validation to evaluate model parameters without looking at the test set. Once model parameters were determined from this internal cross validation procedure, performance was finally evaluated on the previously unseen test set and reported as the mean for the ten folds. Five different BioFINDER test sets were used, using a double cross-validation scheme. External evaluation on ADNI data was performed after all models had been finalized.

Statistically significant differences in demographics were determined using *p*-values from Fisher’s exact test (sex) or t-test for independent samples (remaining variables). To determine statistically significant differences in performance on the test sets for the different models, the results from the 10 folds were used in a Mann-Whitney U-test using the Scipy library (v. 1.4.1) in Python (v. 3.5). All *p*-values less than 0.05 were considered significant. Bootstrapping on the test sets was used to estimate 95% confidence intervals (CI).

## Results

### Cohort characteristics

A total of 332 participants from the Swedish BioFINDER-1 study were included in the present analysis, whereof 223 participants were non-AD participants who were cognitive stable with SCD or MCI for at least four years and the remaining 109 participants were early AD participants with SCD or MCI at baseline who subsequently progressed to AD dementia. As external validation, 108 participants from the ADNI cohort with MCI at baseline were included, whereof 85 participants remained with MCI for at least four years and the remaining 23 participants progressed to AD dementia.

In the BioFINDER cohort, the non-AD participants did not differ from early AD participants significantly on education (12.0 years vs. 11.8 years average, *p*-value 0.72), sex (47.5% female vs. 46.8% female, *p*-value 0.91) or intracranial volume (1136 cm^3^ vs. 1114 cm^3^ average, *p*-value 0.13), but the early AD group had a higher ratio of MCI (71.6% MCI vs. 41.2% MCI, *p*-value < 0.001), was older (72.1 years vs. 70.2 years average, *p*-value < 0.05), had lower baseline MMSE (27.1 vs. 28.2 average, *p*-value < 0.001), higher *APOE* ε4 allele presence (1.0 vs. 0.4, *p*-value < 0.001), worse ADAS score (6.7 vs. 4.2 average, *p*-value < 0.001) and smaller hippocampal volume (2927 mm^3^ vs. 3339 mm^3^ average, *p*-value < 0.001). The same trends could be seen in the ADNI cohort, except that all included participants were MCI.

### Performance for predicting four-year progression to AD

**Fig. 2:**
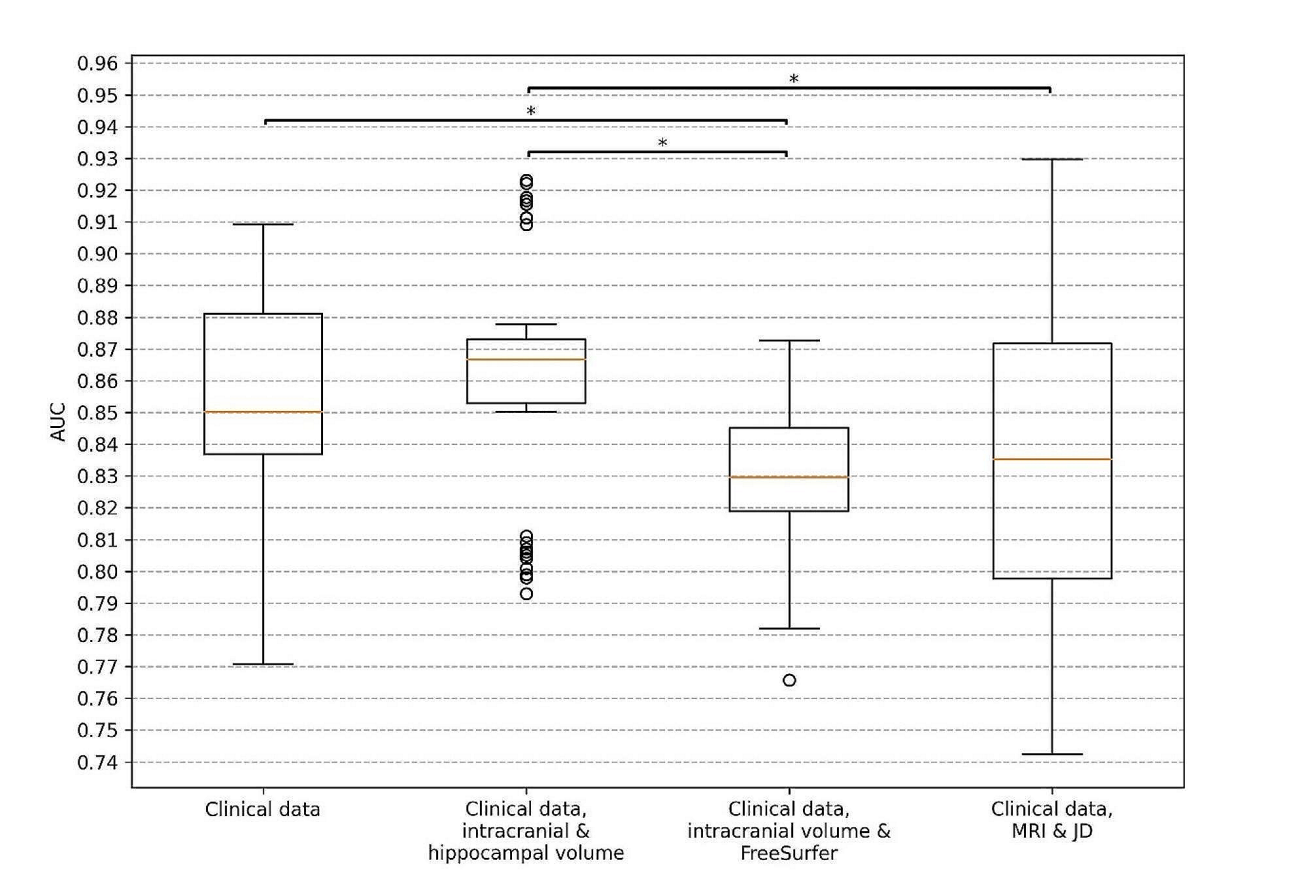
Box plots for AUC when predicting progression into AD dementia within four years. Results are for the BioFINDER test sets using the different models from double cross-validation. The clinical data includes demographics (age, sex, and education), baseline cognition (MMSE score, ADAS delayed word recall) and *APOE* genotype. The orange lines show the median, the boxes span from the first quartile to the third quartile, and the whiskers extend from the box by 1.5 times the interquartile range. Significant differences (*p*-value < 0.05) are indicated with *

**Fig. 3:**
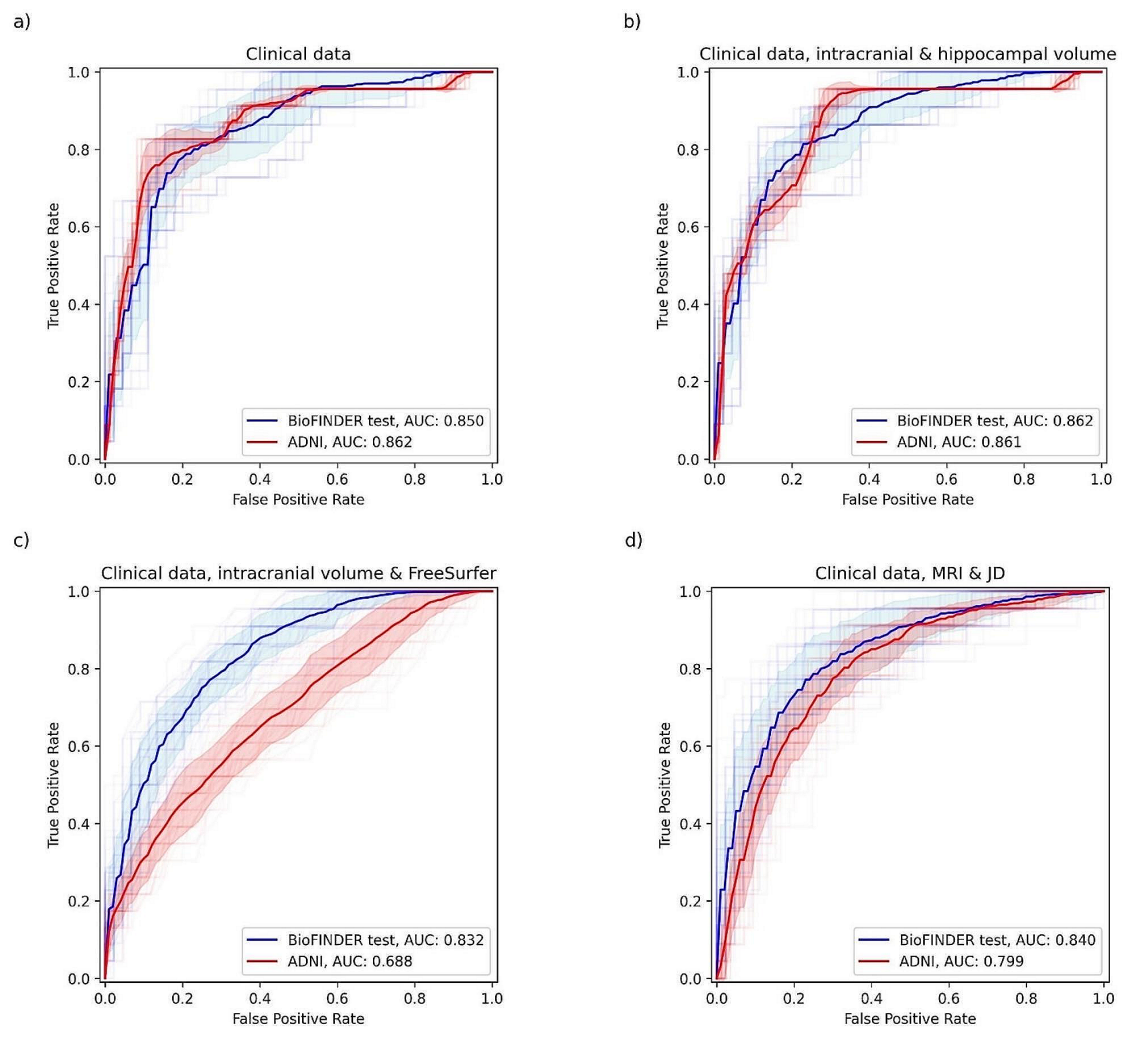
ROC curves and AUC for progression to AD dementia. Models using **a**) clinical data (age, sex, and education, baseline cognition [MMSE score, ADAS delayed word recall] and *APOE* genotype), **b**) clinical data, intracranial volume and hippocampal volume, **c**) clinical data, intracranial volume and FreeSurfer, **d**) clinical data, MRI and JD. The blue curve shows the results on the BioFINDER test data and the red curves the results on the ADNI data. The solid line presents the mean from the five test folds and ten cross-validation folds, the transparent lines present the induvial results, and the shaded areas show the mean plus/minus one standard deviation

The clinical data model consisting of demographics (age, sex, and education), MMSE score, ADAS delayed word recall and *APOE* status had a mean BioFINDER test AUC of 0.850 (CI 0.698–0.954) and MCC of 0.621 (CI 0.427–0.844) for predicting four-year progression to AD dementia. Adding intracranial and hippocampal volume to the clinical data model increased the test AUC to 0.862 (CI 0.728–0.960) and MCC to 0.623 (CI 0.450–0.848). Conversely, adding the FreeSurfer brain region volumes to the clinical data model, the test AUC decreased to 0.832 (CI 0.710–0.929) and MCC to 0.563 (CI 0.416–0.788). Adding whole brain MRI and JD images to the clinical data together in a CNN model led to a decreased test AUC of 0.840 (CI 0.688–0.957) and MCC of 0.605 (CI 0.418 − 0.850). Also, when testing if the differences of the four main models’ performances on the test sets are statistically significant, based on the 50 models from the 5 test sets and 10-fold cross-validation, it was found that the hippocampal volume model was significantly better (*p*-value < 0.05) than both the FreeSurfer and DL models, the Clinical data model was significantly better than the FreeSurfer model, while there was no significant difference between any other pair of models. The results for the external ADNI test data had no significant difference in performance from the BioFINDER test data except for the FreeSurfer model and DL model, which performed significantly worse (FreeSurfer: AUC 0.688, CI 0.536–0.827, DL: AUC 0.799, CI 0.677–0.904). The results are visualized in Figs. 2 and 3 and presented fully in Supplementary Table 1.

### Sensitivity analysis of image modalities and multi-task learning included in deep learning model

The DL model described above included by default both the MRI image and the JD image derived from the image registration procedure. However, extracting JD images represents an extra, more burdensome processing step so we performed a sensitivity analysis in which the effect of fitting the DL model with MRI only or JD only. We found that using the JD image only, which had a mean test AUC of 0.605 (CI 0.418–0.766) for predicting four-year progression to AD dementia, outperformed a model using the MRI image only, which had a mean test AUC of 0.575 (CI 0.274–0.782). Moreover, we found that including both MRI and JD images had a mean test AUC of 0.609 (CI 0.366–0.766), thereby improving on the result from using the JD image only. The multi-task learning approach used was only evaluated for a limited number of $w$-values due to limited computational resources: $w=\text{0,0.025,0.050,0.075,0.01}$. The best value was determined based on the validation data, found to be ${w}_{1}=1,{w}_{2}={w}_{3}=0.025$. All these results are displayed in Supplementary Table 1.

### Performance for predicting four-year decline in cognition

**Fig. 4:**
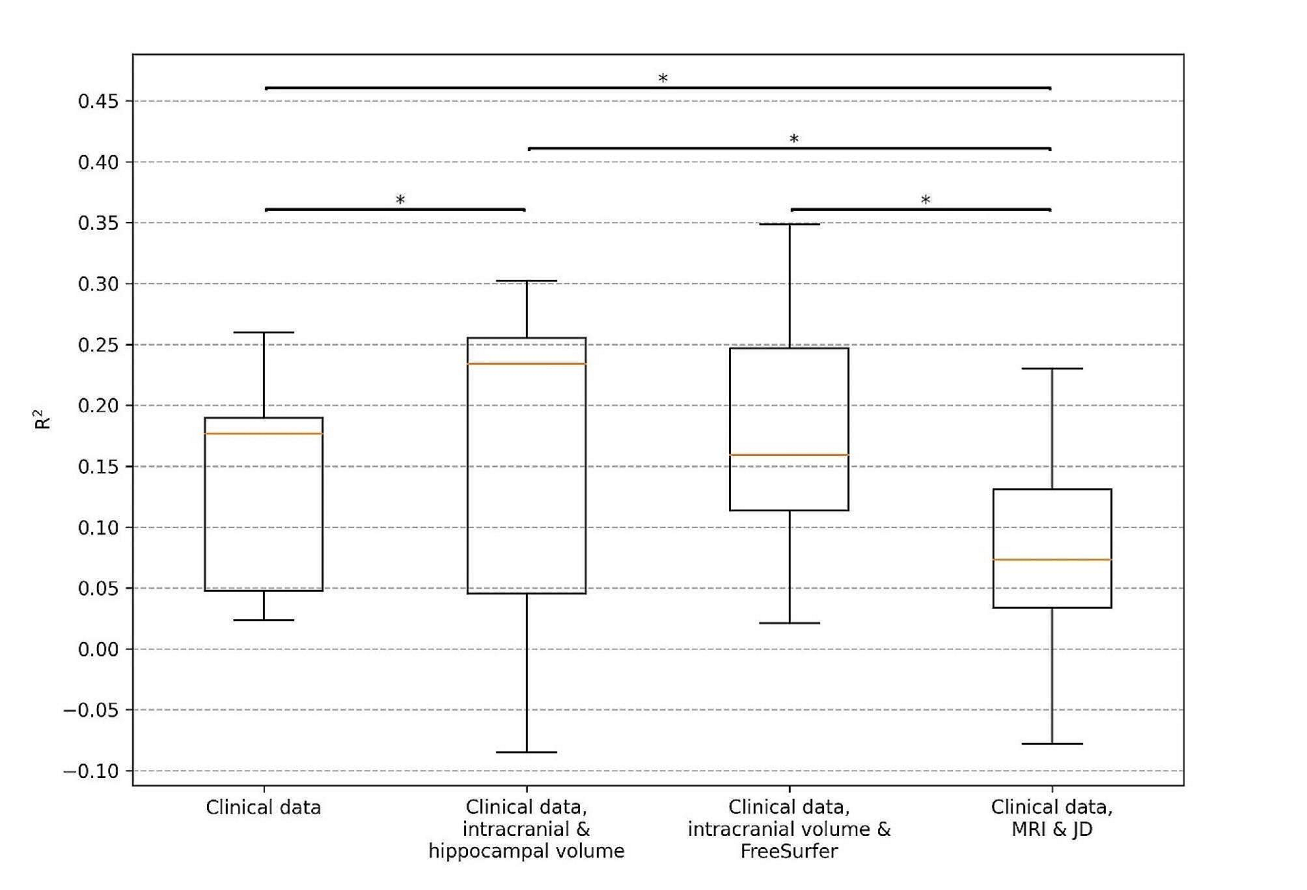
Box plots for R^2^ when predicting four years MMSE slopes. Results are for the BioFINDER test sets using the different models from double cross-validation. The clinical data includes demographics (age, sex, and education), baseline cognition (MMSE score, ADAS delayed word recall) and *APOE* genotype. The orange lines show the median, the boxes span from the first quartile to the third quartile, and the whiskers extend from the box by 1.5 times the interquartile range. Significant differences (*p*-value < 0.05) are indicated with *

**Fig. 5:**
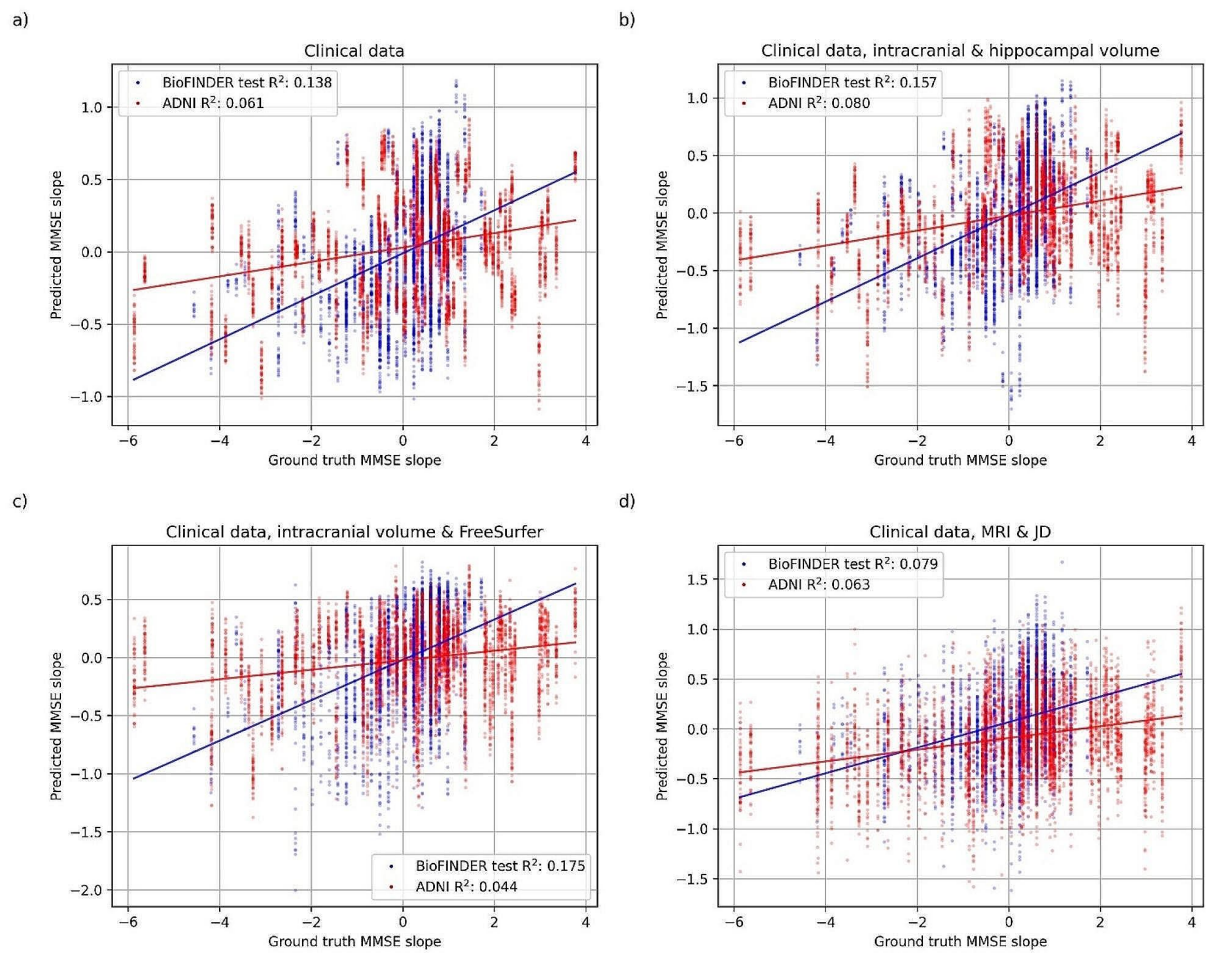
Correlation between predicted slope and corresponding ground truth. Models using **a**) clinical data (age, sex, and education, baseline cognition [MMSE score, ADAS delayed word recall] and *APOE* genotype), **b**) clinical data, intracranial volume and hippocampal volume, **c**) clinical data, intracranial volume and FreeSurfer, **d**) clinical data, MRI and JD. The blue data shows the BioFINDER test sets and the red data shows the external evaluation on ADNI data

The clinical data model consisting of demographics (age, sex, and education), baseline cognition (MMSE score, ADAS delayed word recall) and *APOE* status had a mean BioFINDER test R^2^ of 0.138 (CI -0.171–0.328) for predicting four-year cognitive decline as measured by MMSE. Adding hippocampal volume to the clinical data model improved the mean test R^2^ to 0.157 (CI -0.296–0.403). Adding FreeSurfer brain regions to the clinical data model improved the mean test R^2^ to 0.175 (CI -0.127–0.396). The DL model featuring the clinical data features and the whole brain MRI and JD images had the lowest mean test R^2^ of 0.079 (-0.206–0.267). The values for $w$ that were found optimal for the previous task was used here as well but altered to prioritize this task (${w}_{2}=1,{w}_{1}={w}_{3}=0.025$). All models were significantly better (*p*-value < 0.05) than the DL model, and the hippocampal volume model was furthermore also significantly better than the clinical data model. However, the performance on the external ADNI test data was significantly worse for all models except the DL model. The results are visualized in Figs. 4 and 5 and presented fully in Supplementary Table 2.

### Identifying atrophy patterns from FreeSurfer and deep learning models

**Fig. 6:**
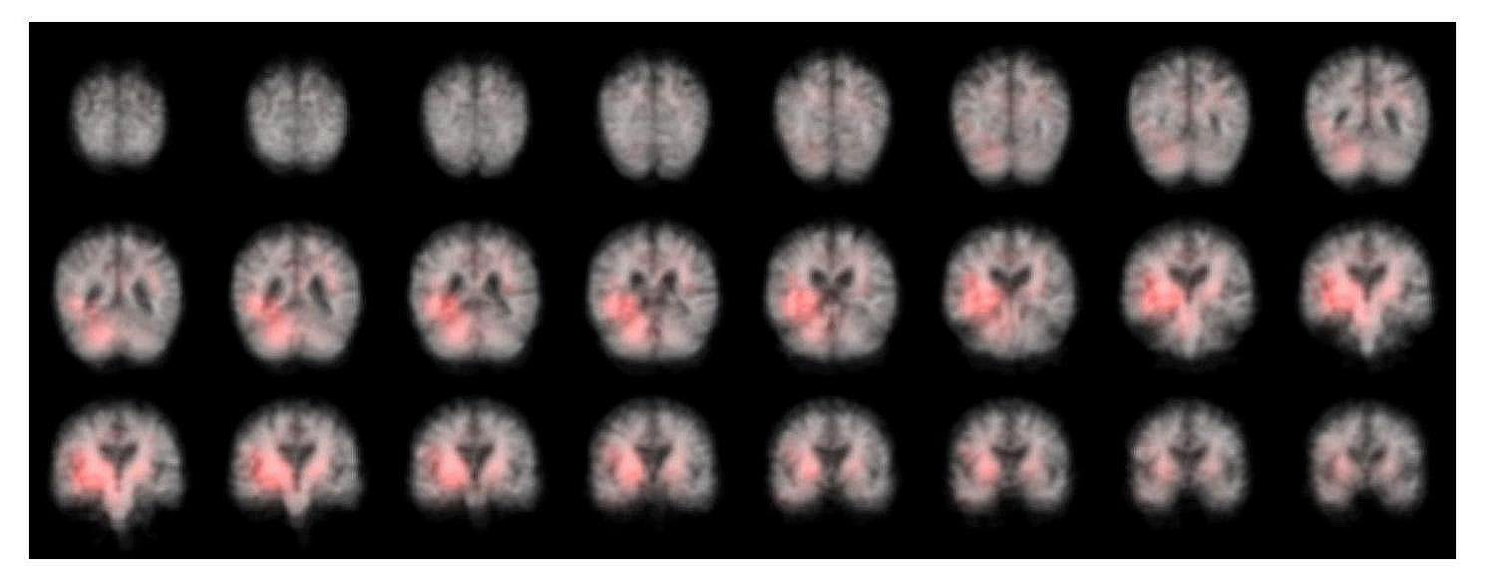
Heatmap with features used by the CNN. This figure shows the results of an occlusion experiment. Areas of the brain which are colored red are those areas whose occlusion led to a drop in model performance compared to when no occlusion was used

Atrophy patterns representing the important brain regions of interest were identified for the DL whole brain model using the patch occlusion method described in the section “Statistical analysis”. Regions in the temporal and parietal lobes were identified, see visualization in Fig. 6.

## Discussion

We developed and evaluated different models for identifying SCD/MCI individuals who are more likely to progress to AD dementia within four years, as well as models for prediction of change in MMSE over four years. The models were based on combinations of demographics, standard cognitive tests, hippocampal volume, volumetric data from FreeSurfer, MRI 3D images and JDs computed from MRI. We focused on realistically evaluating model performances by following a rigorous approach without leakage of information from training to test sets as well as including external evaluation, and we also focused on unbiased comparisons between models of different complexity. In general, we found that a model with only demographics and baseline clinical data (cognitive tests plus *APOE* ε4 status) performed very well, and that only smaller improvements in predictive ability were seen when adding hippocampal volume or volumetrics data from FreeSurfer. We could not see consistent improvements in model performance when using the entire MR images in DL models. Taken together, this suggests that among the predictors tested here, most of the relevant predictive information for patients in the SCD/MCI stage of AD dementia is contained in the baseline cognitive profile together with an MRI assessment of hippocampal atrophy.

The problem of predicting progression to AD dementia has been studied multiple times before, both using MRI but also using data from e.g., PET. While PET has been shown to provide more information than MRI [25], it also has drawbacks such as being expensive and less available wherefore it was not used in this study. MRI in contrast is more readily available and a realistic option for prediction models in clinical practice. Previous publications have reported results with AUC values in the range 0.53–0.98 [25]. However, it is hard to compare the studies in a fair way due to variations in for example imaging modality (e.g., MRI or PET), prediction task (e.g., MCI versus AD, or progressive MCI versus stable MCI with different follow-up times), the dataset used as well as the dataset’s division into development and test data. Our best-performing model with an AUC of 0.86, on both the test data and the external evaluation data, is within the range of previous reported results. Many studies are using data from the publicly available ADNI cohort [40], but due to different exclusion criteria and validation methods the final ADNI datasets vary. Furthermore, according to the review in [12], comparing studies performing classification of AD dementia using DL and MRI, it was found that there may have been data leakage and thus a bias in the reported results in more than half of the surveyed papers. It was shown that when correctly dividing the data such that data from the same subject was never present in both training and test set simultaneously, the accuracy dropped from 99 to 90% [10]. We used data from the Swedish BioFINDER study, which has the major benefit of being a well-characterized cohort in terms of clinical diagnosis and biomarker confirmation of amyloid pathology as well as being more representative to a general memory clinic setting compared to the ADNI cohort (which is a more selected cohort, characterized for example by highly educated study participants, and few vascular co-pathologies). However, as external evaluation we include participants from the ADNI cohort. The test sets were only used for evaluation after all models were finalized, thus not in any way influencing the development of the algorithm or model selection.

Prediction of MMSE slope is a less explored task than prediction of AD dementia. Compared to the binary classification task of separating non-AD and early AD, it has the advantage of being a continuous measure which is potentially less prone to bias and subjectivity compared to a clinical diagnosis. Moreover, cognitive assessment is relevant because it is typically the primary endpoint for clinical trials of AD.

We investigated whether predefined volumetric variables, such as hippocampal volume and volumetric variables from FreeSurfer, or data-driven features from MRI using DL optimized for the given task, provided the most information for prediction of SCD/MCI-to-AD progression and MMSE slope. While the MRI ought to contain at least as much information as the volumetric variables, since the volumetric variables are determined from the MRI, we anyway obtain results showing that the hippocampal volume gives the best prediction for SCD/MCI-to-AD progression while the FreeSurfer variables give the best MMSE slope prediction. The reason for this is probably that the amount of data available for training is too small to learn more representative features using DL and that the model’s capacity is too limited. The limited capacity was chosen to avoid overfitting and define a model suitable to the amount of available data. The results show that more data is needed for DL models to be successful. Thus, the results should not be interpreted as a failure for DL, but rather that its success might require an order of magnitude more data. Furthermore, it has been established for a long time that pathological changes in AD are focused in the hippocampus [41]. A hypothesis for the difference in performance between the DL model and FreeSurfer model is that JDs derived from normalization can be difficult for a severely atrophied brain with very large ventricles. In such cases, native FreeSurfer segmentation could be more informative. However, the DL model also utilizes the MRIs in native space, so the worse performance of the DL model is probably caused by its limited capacity, chosen to fit the number of subjects available.

The DL model used in this work is based on the network developed by [14], which uses parameter-efficient layers such as grouped and separable convolutions. However, a few modifications were done to optimize it for our settings. Similar to the original network we used multi-task learning, but for other tasks. Instead of simultaneously predict SCD/MCI-to-AD progression as well as classify AD dementia vs. healthy controls using a dataset with normal, stable MCI and progressive MCI cases, we use a dataset with only SCD/MCI cases and predict SCD/MCI-to-AD progression together with four years MMSE slopes and hippocampal volume. We show that the multi-task learning approach improves the performance (Supplementary Table 1). Training the model for all tasks simultaneously has a regularizing effect on the training and reduces the risk of overfitting the model to the relatively small dataset. Furthermore, it is likely that different tasks find descriptive information in similar features, hence the multi-task learning should not limit the models’ capacities.

Our best-performing model for prediction of SCD/MCI-to-AD progression utilizes the clinical data together with the hippocampal volume. However, the clinical data model alone performs very well and, in most cases, outperforms the other models which only utilizes one type of data (hippocampal volume, FreeSurfer variables, MRI or JD), Supplementary Table 1. A similar result was obtained in the study by [14], where they obtained an AUC of 0.79 when using MRI only and an AUC of 0.88 when using clinical variables only, similarly to our clinical data model. However, in the study by [22], they received significantly better results using a DL model and MRI images compared to e.g. MMSE, hippocampal volume, cerebrospinal fluid Aβ and tau. They did however not evaluate using multiple simple features combined or looked at longitudinal cognitive decline using continuous measures of cognition, as we have done in this study.

While the atrophy patterns identified as important by the DL model differ for all patients, we can observe that the regions often coincide with regions that have been previously associated with AD dementia such as temporal and parietal lobes, see Fig. 6. The finding that the regions identified as important varied across patients is inherent to DL models compared to statistical models such as logistic regression that instead identify a common pattern of atrophy in the entire study population. The individualized nature of atrophy patterns derived from DL is a strong benefit because it can allow for personalized explanations as to how an individual’s predicted risk score was derived.

The main limitation of our study is the size of the dataset used, although it is similar in size (*N* = 332) to what has been used in some previous studies [ (11): *N* = 968, (14): *N* = 785, (15): *N* = 509, (16): *N* = 559, (20): *N* = 200, (21): *N* = 819, (22): *N* = 786] but of higher quality with fewer healthy controls and AD dementia and higher ratio of SCD/MCI patients [our: *N* = 332, (11): *N* = 413, (14): *N* = 409, (15): *N* = 210, (16): *N* = 216, (20): *N* = 97, (21): *N* = 398, (22): *N* = 582]. Another limitation is the selection of model architecture and hyperparameters, which can be further explored. We investigated different amount of multi-task learning (Supplementary Table 1) and different techniques for normalization of the input data, but further optimization could be done. However, the network architecture used has been optimized in previous studies using another dataset [13, 14], and by not optimizing it further we reduce the risk of overfitting it to our study. Finally, the evaluation is limited by the gold standard, which is determined by the cognition variables and thus is biased towards those. Thus, it is not surprising that these variables have high correlation with the outcomes we use. The clinical gold standard is used clinically today and there is no similar metric that is based on for example volumetric features. However, it could be that such a metric also has a high correlation with both patients’ symptoms as well as our MRI based models. Another aspect of our work is that we did not test for conversion to all-cause dementia. Our aim was to develop tools that specifically predicted development of dementia due to AD. Due to the recent breakthroughs in DMTs against AD [7, 8], it is of high importance to specifically predict future development of dementia due to AD rather than dementia due to other diagnoses, since only patients at risk for developing dementia due to AD should receive these new types of treatment.

## Conclusions

We developed and evaluated four different models on two different outcomes. The models perform similar, but the clinical data model using only demographics (gender, age, education), baseline cognition (MMSE score, ADAS delayed word recall) and *APOE* status performs well, and only small improvements can be seen when adding hippocampal volume or regional MRI gray matter volumes extracted using FreeSurfer. For identification of patients with high risk of SCD/MCI-to-AD progression within four years we obtained an AUC of 0.862 and for four-years MMSE slope prediction we obtained an R^2^ score of 0.157 using clinical data and hippocampal volume. Similar results for prediction of SCD/MCI-to-AD progression was seen in the external ADNI cohort, while the results for predicting four-years MMSE slope was significantly deteriorated. The result for SCD/MCI-to-AD progression is similar to previous studies, while to the best of our knowledge no data are currently available with respect to prediction of MMSE slope. The best DL models identified uses multi-task learning, by being trained to simultaneously predict both SCD/MCI-to-AD progression, four years MMSE slope as well as hippocampal volume. We confirmed that the areas found as interesting by the DL models are reasonable using an occlusion algorithm. The results are humbling with respect to what can be achieved by DL models. In the future, it may be tested if better performance can be achieved by increasing the training sample size, or by adding additional investigational modalities or MRI-sequences, or by fine-tuning the outcome measures to minimize noise and variability.

### Electronic supplementary material

Below is the link to the electronic supplementary material.

Supplementary Material 1

## Data Availability

Anonymized data can be shared to qualified academic researchers after request for the purpose of replicating procedures and results presented in the study. Data transfer must be performed in agreement with EU legislation regarding general data protection regulation and decisions by the Ethical Review Board of Sweden and Region Skåne.

## Abbreviations

AD: Alzheimer’s disease
ADAS: Alzheimer’s Disease Assessment Scale
ADNI: Alzheimer’s Disease Neuroimaging Initiative
AI: Artificial intelligence
AUC: Area under the curve
CI: Confidence intervals
CNN: Convolutional neural network
DL: Deep learning
DMT: Disease–modifying treatments
JD: Jacobian determinant
MCC: Matthes correlation coefficient
MCI: Mild cognitive impairment
MMSE: Mini Mental State Examination
MRI: Magnetic resonance imaging
SCD: Subjective cognitive decline
