# Supplementary material for: Comparing a pre-defined versus deep learning approach for extracting brain atrophy patterns to predict cognitive decline due to Alzheimer’s disease in patients with mild cognitive symptoms
Source: Alzheimers Res Ther. 2024 Mar 19;16:61. doi: 10.1186/s13195-024-01428-5 (PMC10949809; doi:10.1186/s13195-024-01428-5)
Supplement: Supplementary file 1 — Supplementary Material 1 [file 13195_2024_1428_MOESM1_ESM.pdf]

Supplementary

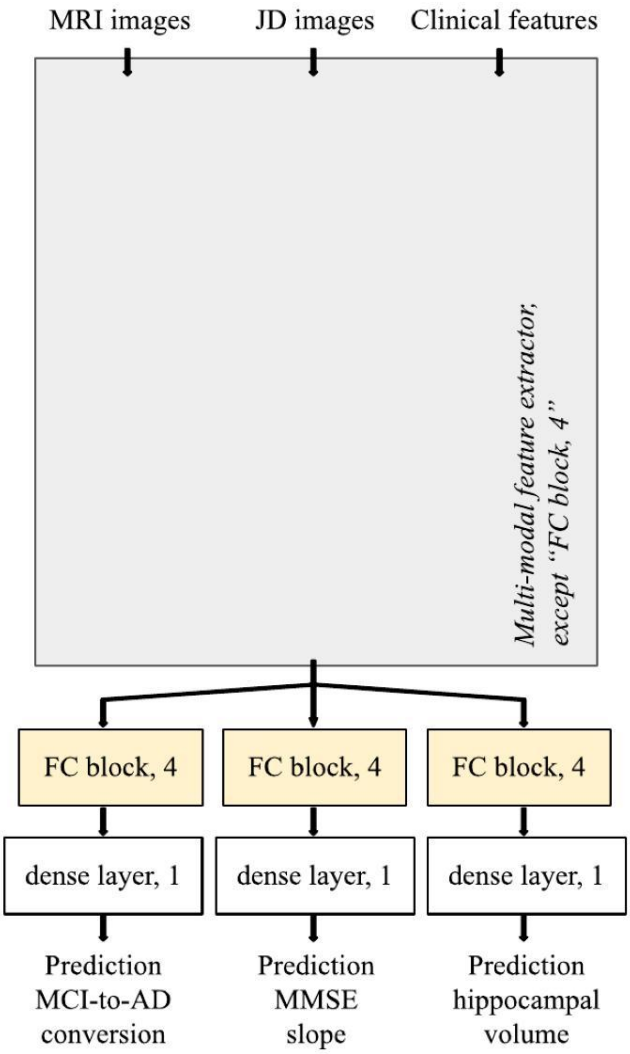

Supplementary Figure 1. Illustration of the CNN architecture. The CNN was trained for prediction of SCD/MCI-to-AD progression as well as prediction of four years MMSE slope. The network is based on the multi-modal feature extractor first presented in (14) but with modified final layers. The same notations as in (14) are used for the final layers. The network is trained with a combined loss function, using a weighted sum of the loss for each output type, using multi-task learning.

Supplementary Table 1. AUC and MCC for the different models when predicting progression into AD dementia within four years.

Input	Method	BioFINDER Test AUC	BioFINDER Test MCC	ADNI Test AUC	ADNI Test MCC
**MRI**	CNN *, $w_1 = 1$, $w_2, w_3 = 0$	0.575 (0.274 - 0.782)			
**JD**	CNN *, $w_1 = 1$, $w_2, w_3 = 0$	0.605 (0.418 - 0.766)			
**MRI + JD**	CNN*, $w_1 = 1$, $w_2, w_3 = 0$	0.609 (0.366 - 0.766)			
**MRI + JD**	CNN *, $w_1 = 1$, $w_2, w_3 = 2.5e^{-2}$	0.626 (0.486 - 0.762)			
**MRI + JD**	CNN *, $w_1 = 1$, $w_2, w_3 = 5e^{-2}$	0.639 (0.499 - 0.773)			
**MRI + JD**	CNN *, $w_1 = 1$, $w_2, w_3 = 7.5e^{-2}$	0.651 (0.506 - 0.791)			
**MRI + JD**	CNN *, $w_1 = 1$, $w_2, w_3 = 1e^{-1}$	0.644 (0.507 - 0.773)			
**Clinical data, only demographics**	Logistic regression	0.588 (0.417 - 0.745)	0.291 (0.127 - 0.527)	0.631 (0.512 - 0.742)	0.266 (0.192 - 0.431)
**Clinical data, w/o demographics**	Logistic regression	0.841 (0.676 - 0.953)	0.579 (0.386 - 0.840)	0.823 (0.703 - 0.922)	0.514 (0.387 - 0.720)
**Intracranial vol.**	Logistic regression	0.530 (0.364 - 0.689)	0.215 (0.101 - 0.434)	0.630 (0.491 - 0.761)	0.233 (0.144 - 0.479)
**Hippocampal vol.**	Logistic regression	0.752 (0.607 - 0.873)	0.474 (0.344 - 0.708)	0.760 (0.645 - 0.862)	0.379 (0.285 - 0.598)
**FreeSurfer**	Random forest	0.729 (0.574 - 0.865)	0.432 (0.279 - 0.685)	0.499 (0.339 - 0.660)	0.212 (0.050 - 0.462)
**Clinical data**	Logistic regression	0.850 (0.698 - 0.954)	0.621 (0.427 - 0.844)	0.862 (0.755 - 0.947)	0.629 (0.460 - 0.823)
**Clinical data + Intracranial vol. + Hippocampal vol.**	Logistic regression	0.862 (0.728 - 0.960)	0.623 (0.450 - 0.848)	0.861 (0.759 - 0.941)	0.565 (0.464 - 0.766)
**Clinical data + Intracranial vol. + FreeSurfer**	Random forest	0.832 (0.710 - 0.929)	0.563 (0.416 - 0.788)	0.688 (0.536 - 0.827)	0.343 (0.217 - 0.585)
**Clinical data + MRI + JD**	CNN, $w_1 = 1$, $w_2, w_3 = 2.5e^{-2}$	0.840 (0.688 - 0.957)	0.605 (0.418 - 0.850)	0.799 (0.677 - 0.904)	0.468 (0.325 - 0.696)

Results from all models and both BioFINDER and ADNI test data. The variables w_1, w_2, w_3 for the CNN refer to the weighting of the loss function, compare with equation (1). The DL models denoted with * where only trained and evaluated for one test fold (thus, 10-fold cross-validation was done once), due to the training of each such model took several days. The best performing DL model was later trained together with the clinical data for all five test folds. The clinical data includes demographics (age, sex, and education), baseline cognition (MMSE score, ADAS delayed word recall) and *APOE* genotype. The parentheses show 95% confidence intervals.

Supplementary Table 2. R^2 for the different models when predicting progression into AD dementia within four years.

Input	Method	BioFINDER Test R^2	ADNI Test R^2
MRI	CNN, $w_2 = 1$, $w_1, w_3 = 0$ *	-0.012 (-0.118 - 0.058)	
JD	CNN, $w_2 = 1$, $w_1, w_3 = 0$ *	-0.017 (-0.206 - 0.113)	
MRI + JD	CNN, $w_2 = 1$, $w_1, w_3 = 0$ *	0.010 (-0.149 - 0.130)	
MRI + JD	CNN, $w_2 = 1$, $w_1, w_3 = 2.5e^{-2}$ *	0.029 (-0.106 - 0.129)	
Clinical data, only demographics	Linear regression	0.003 (-0.147 - 0.060)	-0.006 (-0.070 - 0.022)
Clinical data, w/o demographics	Linear regression	0.148 (-0.148 - 0.339)	0.082 (-0.016 - 0.137)
Intracranial vol.	Linear regression	-0.005 (-0.128 - 0.032)	-0.028 (-0.166 - 0.005)
Hippocampal vol.	Linear regression	0.076 (-0.150 - 0.205)	0.064 (-0.001 - 0.113)
FreeSurfer	Random forest	0.120 (-0.213 - 0.344)	-0.013 (-0.128 - 0.063)
Clinical data	Linear regression	0.138 (-0.171 - 0.328)	0.061 (-0.050 - 0.126)
Clinical data + Intracranial vol. + Hippocampal vol.	Linear regression	0.157 (-0.296 - 0.403)	0.080 (-0.071 - 0.158)
Clinical data + Intracranial vol. + FreeSurfer	Random forest	0.175 (-0.127 - 0.396)	0.044 (-0.079 - 0.126)
Clinical data + MRI + JD	CNN, $w_2 = 1$, $w_1, w_3 = 2.5e^{-2}$	0.079 (-0.206 - 0.267)	0.063 (-0.090 - 0.157)

Results from all models and both BioFINDER and ADNI test data. The variables w_1, w_2, w_3 for the CNN refer to the weighting of the loss function, compare with equation (1). The DL models denoted with * where only trained and evaluated for one test fold (thus, 10-fold cross-validation was done once), due to the training of each such model took several days. The best performing DL model was later trained together with the clinical data for all five test folds. The clinical data includes demographics (age, sex, and education), baseline cognition (MMSE score, ADAS delayed word recall) and *APOE* genotype. The parentheses show 95% confidence intervals.

The 68 FreeSurfer variables:

aparc_grayvol_bankssts_L; aparc_grayvol_bankssts_R; aparc_grayvol_caudalanteriorcingulate_L; aparc_grayvol_caudalanteriorcingulate_R; aparc_grayvol_caudalmiddlefrontal_L; aparc_grayvol_caudalmiddlefrontal_R; aparc_grayvol_cuneus_L; aparc_grayvol_cuneus_R; aparc_grayvol_entorhinal_L; aparc_grayvol_entorhinal_R; aparc_grayvol_frontalpole_L; aparc_grayvol_frontalpole_R; aparc_grayvol_fusiform_L; aparc_grayvol_fusiform_R; aparc_grayvol_inferiorparietal_L; aparc_grayvol_inferiorparietal_R; aparc_grayvol_inferiortemporal_L; aparc_grayvol_inferiortemporal_R; aparc_grayvol_insula_L; aparc_grayvol_insula_R; aparc_grayvol_isthmuscingulate_L; aparc_grayvol_isthmuscingulate_R; aparc_grayvol_lateraloccipital_L; aparc_grayvol_lateraloccipital_R; aparc_grayvol_lateralorbitofrontal_L; aparc_grayvol_lateralorbitofrontal_R; aparc_grayvol_lingual_L; aparc_grayvol_lingual_R; aparc_grayvol_medialorbitofrontal_L; aparc_grayvol_medialorbitofrontal_R; aparc_grayvol_middletemporal_L; aparc_grayvol_middletemporal_R; aparc_grayvol_paracentral_L; aparc_grayvol_paracentral_R; aparc_grayvol_parahippocampal_L; aparc_grayvol_parahippocampal_R; aparc_grayvol_parsopercularis_L; aparc_grayvol_parsopercularis_R; aparc_grayvol_parsorbitalis_L; aparc_grayvol_parsorbitalis_R; aparc_grayvol_parstriangularis_L; aparc_grayvol_parstriangularis_R; aparc_grayvol_pericalcarine_L; aparc_grayvol_pericalcarine_R; aparc_grayvol_postcentral_L; aparc_grayvol_posteriorcingulate_L; aparc_grayvol_posteriorcingulate_R; aparc_grayvol_precentral_L; aparc_grayvol_precentral_R; aparc_grayvol_precuneus_L; aparc_grayvol_precuneus_R; aparc_grayvol_rostralanteriorcingulate_L; aparc_grayvol_rostralanteriorcingulate_R; aparc_grayvol_rostralmiddlefrontal_L; aparc_grayvol_rostralmiddlefrontal_R; aparc_grayvol_superiorfrontal_L; aparc_grayvol_superiorfrontal_R; aparc_grayvol_superiorparietal_L; aparc_grayvol_superiorparietal_R; aparc_grayvol_superiortemporal_L; aparc_grayvol_superiortemporal_R; aparc_grayvol_supramarginal_L; aparc_grayvol_supramarginal_R; aparc_grayvol_temporalpole_L; aparc_grayvol_temporalpole_R; aparc_grayvol_transversetemporal_L; aparc_grayvol_transversetemporal_R
